# Kunden umfassend kennenlernen – Erfahrungen einer Shadowing-Studie an einem internationalen Verkehrsflughafen

**DOI:** 10.1365/s35764-021-00337-8

**Published:** 2021-04-26

**Authors:** Henner Gimpel, Thomas Hinterholzer, Julia Lanzl, Torsten Marheineke, Fabiola Pfauser, Maximilian Röglinger

**Affiliations:** 1 Kernkompetenzzentrum Finanz- & Informationsmanagement, Universität Augsburg, Augsburg, Deutschland; 2 Projektgruppe Wirtschaftsinformatik des Fraunhofer FIT, Universität Augsburg, Augsburg, Deutschland; 3 Universität Hohenheim, Stuttgart, Deutschland; 4 Flughafen München GmbH, München, Deutschland; 5 Kernkompetenzzentrum Finanz- & Informationsmanagement, Universität Bayreuth, Bayreuth, Deutschland

Flughäfen sehen sich einem zunehmenden Wettbewerb gegenüber. Auch der Flughafen München als Europas erster und einziger 5‑Star-Airport kann seine Vorreiterstellung nur erhalten, wenn er konsequent eine kundenzentrierte Strategie verfolgt und höchstmögliche Qualitätsstandards sowie Serviceorientierung für seine Kunden erfüllt. Die vorliegende Arbeit zeigt die Erfahrungen bei der Durchführung einer Shadowing-Studie am Flughafen München. Ergänzend zu quantitativen Kundenanalysen zeigt sich Shadowing als effektiv, um die tatsächliche Passenger Journey zu identifizieren sowie Kundenerlebnisse, Herausforderungen und Bedürfnisse zu analysieren. Auf Basis der Ergebnisse können gezielte Handlungsmaßnahmen abgeleitet werden, um die Kunden zu begeistern. Die Chancen aus dem Shadowing bestehen dabei nicht nur für Flughafenbetreiber, sondern lassen sich auch auf andere Anwendungsbereiche im Dienstleistungssektor übertragen.

## Zusammenfassung

- Flughäfen stehen in einem immer intensiveren Wettbewerb mit andern Flughäfen und anderen Mobilitätsdienstleistern und müssen daher die Kundenzufriedenheit in den Vordergrund stellen.
- Gemeinsam mit dem Flughafen München wurde erstmals eine Shadowing-Studie mit Fokus auf das Kundenerlebnis durchgeführt.
- Durch das Begleiten der Passagiere auf ihrer „Reise“ durch den Flughafen konnten wichtige Erkenntnisse erlangt und Maßnahmen zur Verbesserung der Kundenzufriedenheit abgeleitet werden.

## Kernthesen

- Quantitative Befragungen allein reichen nicht aus, um das Erlebnis von Passagieren umfassend zu verstehen.
- Die Shadowing-Methode ist geeignet, um umfassende Einblicke in die Wegefindung und Emotionen der Kunden zu erlangen – nicht nur im Luftfahrtsektor.

## Handlungsempfehlungen

- Flughäfen müssen zur Erreichung einer höchstmöglichen Kundenzufriedenheit verstärkt auf ihre Passagiere zugehen, um die konkreten Bedürfnisse zu verstehen.
- Um dem hohen Aufwand einer Shadowing-Studie zu begegnen, sollten Unternehmen die Untersuchung auf konkrete Fragestellungen und Kundensegmente eingrenzen.
- In die Vorbereitung und Durchführung des Shadowings müssen alle Fachbereiche eingebunden werden, um hohe Relevanz und Akzeptanz der Ergebnisse zu erreichen.

Durch immer mehr Konkurrenz insbesondere im außereuropäischen Bereich, die zunehmende Bedeutung des Non-Aviation-Geschäfts und in Zeiten nach der COVID-19-Pandemie potenziell rückläufigen Flugaufkommens stehen Flughäfen unter einem immer höheren Wettbewerbsdruck. Eine zentrale Herausforderung für Flughäfen besteht dabei in den immer anspruchsvolleren Kunden und den sich schneller ändernden Anforderungen. Darüber hinaus führen digitale Technologien wie Social Media, Mobile Apps und das Internet der Dinge zu einem tiefgreifenden Wandel der Gesellschaft und einem neuen Selbstverständnis der Kunden. Kunden sind vernetzt, informiert und selbstbewusst. Sie erwarten eine individuelle Behandlung, hohe Qualität und Transparenz sowie innovative Dienstleistungen auf Basis digitaler Technologien. Ohne eine konsequente Orientierung am Endkunden kann im digitalen Zeitalter kaum ein Unternehmen im Wettbewerb bestehen [1]. Hochqualitative Produkte und Dienstleistungen reichen nicht mehr aus. Vielmehr gilt es, Kunden in allen Segmenten über alle Kontaktpunkte und Interaktionskanäle hinweg ein herausragendes Kundenerlebnis zu bieten. Nur so kann aus dem einmaligen Kundenerlebnis langfristig Kundenloyalität und -bindung sowie ökonomischer Erfolg generiert werden [3]. Diese Notwendigkeit der Kundenzentrierung wird an Flughäfen insbesondere auch dadurch immer wichtiger, dass Flughäfen als Infrastrukturbetreiber heute nur noch gut die Hälfte ihres Umsatzes durch das klassische Aviation-Geschäft machen. Die andere Hälfte kommt aus dem Non-Aviation-Bereich – beispielsweise der Gastronomie und dem Einzelhandel – , in dem Kunden während ihres Aufenthalts am Flughafen eine noch zentralere Rolle für den Flughafen einnehmen.

## Fehlende direkte Kundenbeziehung erschwert Einblicke in die Kundenbedürfnisse

Die Analyse und Gestaltung des Kundenerlebnisses ist ein kritischer Erfolgsfaktor, um langfristig profitable Kundenbeziehungen aufzubauen und zu erhalten. Inzwischen haben viele Flughäfen verstanden, dass der Aufbau von Kundenbeziehungen immer wichtiger wird. In der Entwicklung hin zu einer kundenorientierteren Strategie stehen Flughäfen jedoch vor einer zentralen Herausforderung: Der Kontakt mit den Passagieren bzw. Endkunden besteht vielmehr durch die Airlines und nicht durch den Flughafen selbst als Infrastrukturbetreiber [2].

Die Digitalisierung bringt dafür Chancen mit sich. Durch digitale Technologien können eigene Kontaktpunkte auf der Passenger Journey, die im Fall der Kunden eines Flughafens oftmals eine wirkliche Reise darstellt, geknüpft werden. So kann der erste Kontakt schon vor dem Besuch des Flughafens sein, wenn sich Passagiere über die Flughafen-Website über die dortigen Abläufe, Parkmöglichkeiten und Services informieren. Über digitale Info-Terminals oder Flughafen-Apps können sich Passagiere während ihres Aufenthalts jederzeit über die aktuellen Gegebenheiten und Angebote am Flughafen informieren und somit die Beziehung weiter ausbauen. Durch eine Abstimmung der dargestellten Informationen auf verschiedene Passagiergruppen kann über diese Wege dem Passagier eine gezielte und situationsspezifische Unterstützung auf seiner Reise durch den Flughafen gegeben werden.

Beim Ausbau digitaler Kontaktpunkte darf allerdings nicht der Fokus auf die analoge Passenger Journey verloren gehen, sondern es müssen sowohl die digitale als auch die physische Welt im Rahmen einer Omnikanal-Strategie berücksichtigt werden, sodass die Prozesse für Passagiere bestmöglich gestaltet sind und zusätzlich über digitale Technologien Möglichkeiten der Personalisierung und der Echtzeitfähigkeit genutzt werden. Durch eine Abstimmung der vielen Möglichkeiten kann das Kundenerlebnis weiter verbessert werden.

Insgesamt gilt: Bevor ein Unternehmen Kundenerlebnisse managen kann, muss es diese verstehen, insbesondere indem es die Erfahrungen und Erlebnisse der Kunden misst sowie emotionale Bindungsfaktoren identifiziert. Wir sprechen in diesem Zusammenhang nicht mehr nur von Touchpoints, sondern von Experience Points. Bislang greifen Flughäfen in dem Versuch, Kundenbedürfnisse zu verstehen, zu verschiedenen klassischen sowie innovativen Methoden und setzen auf quantitative Kundenbefragungen oder digitale Feedback-Terminals mit einer schnellen Einschätzung des Erlebnisses anhand von Smileys. Hierbei werden jedoch oftmals nur Abschnitte der Passenger Journey, wie zum Beispiel der Toilettenbesuch, bewertet. Es fehlt an der notwendigen Nähe, um den Passagier vollumfänglich auf seiner Passenger Journey zu verstehen. Um noch mehr und genauere Erkenntnisse über die Passagiere zu erhalten, sind Flughäfen ununterbrochen auf der Suche nach neuen Methoden, um dem Anspruch an höchste Qualität und Innovation gerecht zu werden. Mit dem Einsatz des Shadowings wurde erstmals eine neue Methode für die Erlangung eines umfassenden Verständnisses der Kundenbedürfnisse am Flughafen München eingesetzt. Dadurch konnten die etablierten quantitativen Maßnahmen um wichtige Erkenntnisse aus der qualitativen Herangehensweise des Shadowings ergänzt werden.

## Shadowing am Flughafen München

Als erster in Europa hat der Flughafen München im Frühjahr 2015 die 5‑Star-Airport-Auszeichnung des Luftfahrtforschungsinstituts Skytrax für seine erstklassige Servicequalität erhalten und bis heute aufrechterhalten. Diese weltweit nur an 14 Flughäfen vergebene Auszeichnung bestätigt, dass sowohl Urlauber als auch Geschäftsreisende während ihres Aufenthalts am Flughafen München erstklassiges Ambiente und Komfort, ein vielfältiges Serviceangebot, optimierte Abläufe, eine einfache Orientierung sowie eine einzigartige Gastfreundschaft erleben dürfen. Das Markenversprechen „Verbindung leben“ und ein konsequent umgesetzter Dienstleistungsgedanke stehen für den Flughafen München an erster Stelle. Mit der Auszeichnung zum 5‑Star-Airport geht daher das Bestreben einher, das erreichte Qualitätsniveau zu halten und auszubauen. So ist es der eigene Anspruch des Flughafens München, „Der Beste unter den Großen“ zu sein.

Ziel des Flughafens München beim Einsatz der Shadowing-Methode war es, Passagiere während ihrer Journey am Flughafen genauer zu verstehen. Zentrale Fragestellungen waren die tatsächlich zurückgelegten Wege, Herausforderungen für Passagiere und begeisternde Elemente sowie die Annahme von digitalen Touchpoints wie beispielsweise Check-in-Automaten oder die elektronische Passkontrolle. Dabei sollte insbesondere auch die Emotion der Passagiere – positiv wie negativ – eingefangen werden, um auf Basis der Ergebnisse das Erlebnis der Passagiere noch besser zu gestalten.

Shadowing ist eine qualitative Forschungsmethode, bei der eine untersuchende Person (Shadower) die zu untersuchende Person (zum Beispiel Mitarbeiter eines Unternehmens oder Kunden) über einen längeren Zeitraum begleitet und beobachtet [4]. Der Vorteil des Shadowings gegenüber anderen qualitativen Forschungsmethoden liegt in einem wesentlich höheren Detailgrad sowie einer größeren Vollständigkeit der gesammelten Daten über eine individuelle Person und deren Verhaltensweisen [4]. McDonald (2005) nennt eine Reihe an Best Practices, die in der Vorbereitung und Durchführung des Shadowings beachtet werden sollten. So ist es beispielsweise während des Shadowings enorm wichtig, alle Beobachtungen in Bezug auf Handlung, Mimik, Gestik und Emotionen der beobachteten Person zu dokumentieren. Dafür wurden den Shadowern im Rahmen der Untersuchung am Flughafen München Templates zur Verfügung gestellt, auf welchen die Orte am Flughafen einer (Teil‑)Beobachtung, die Handlung der Passagiere sowie die beobachteten Emotionen möglichst schnell und umfangreich dokumentiert werden konnten. Zudem war es für alle Shadower vor Beginn des Projekts unerlässlich, sich intensiv mit dem Flughafen und der Umgebung dort sowie den Charakteristiken verschiedener Passagiergruppen auseinanderzusetzen, um die Handlungen der Passagiere jederzeit korrekt einordnen zu können.

Das Shadowing-Projekt am Flughafen München konzentrierte sich auf drei Passagiergruppen (siehe Tab. 1): Geschäftsreisende, privat reisende Einzelpersonen oder Paare sowie Familien. In der Regel kennen Geschäftsreisende den Flughafen und die Prozesse auf der einen Seite sehr gut und haben daher keine großen Probleme, sich zurechtzufinden. Auf der anderen Seite haben sie hohe Erwartungen an einen reibungslosen Ablauf. Privatreisende sowie Familien haben tendenziell weniger Erfahrung mit Flughäfen, fühlen sich daher unsicherer in den Abläufen und benötigen eine bestmögliche Unterstützung durch die verschiedenen Kommunikationskanäle. Familien stehen neben den ohnehin schon aufwendigen Abläufen vor der zusätzlichen Herausforderung, sperriges Gepäck wie beispielsweise Kinderwägen durch die Sicherheitskontrolle schleusen und gleichzeitig dafür sorgen zu müssen, Kinder zu beschäftigen oder zu beruhigen.

**Table 1:** 

		Geschäftsreisende	Privatreisende (ohne Familien)	Familien
Insgesamt		90	88	14
Einstiegsart	Abfliegende	48	53	7
	Umsteigende	24	20	2
	Ankommende	18	15	5
Nationalität	Deutsch	67	57	8
	Nicht deutsch	23	31	6

Das Shadowing lief wie folgt ab: Die Shadower warteten an verschiedenen Ankunftsorten am Flughafen (zum Beispiel S‑Bahnhof, Gates von ankommenden Flügen), sprachen zufällig ausgewählte Passagiere an und befragten sie zu ihrer Teilnahmebereitschaft am Shadowing. Nachdem der Passagier in die Teilnahme eingewilligt hatte, folgte ihm ein Shadower auf dem gesamten Weg am Flughafen mit Abstand, um den Passagier nicht zu beeinflussen. Währenddessen protokollierte der Shadower möglichst viele Informationen wie den Weg, Emotionen, welche anhand der Mimik und Gestik abgelesen werden konnten, sowie Stellen, an denen Passagiere Schwierigkeiten bei der Orientierung hatten und sich lange mit der Beschilderung beschäftigten. Am Ende des Weges wurden die Passagiere zu ihren Erlebnissen im Rahmen eines kurzen semistrukturierten Interviews befragt.

## Ergebnisse

Während der Studie konnten 192 Passenger Journeys dokumentiert und ausgewertet werden. Tab. 1 zeigt die Aufteilung auf die verschiedenen Zielgruppen.

**Figure 1:**
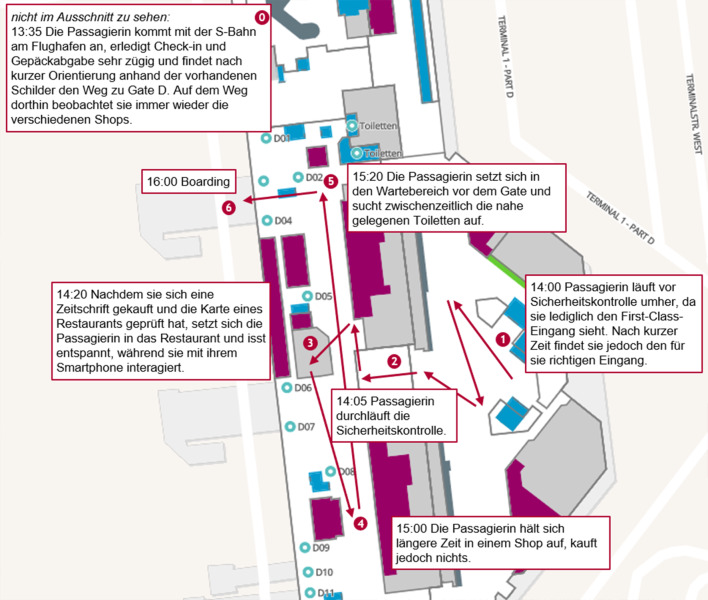

Abb. 1 zeigt beispielhaft einen Ausschnitt des Weges einer Passagierin, die sich anhand der Beschilderung am Terminal 1 nach kurzer Zeit gut zurecht finden konnte. Nachdem sie die notwendigen Prozessschritte des Check-ins der Gepäckabgabe und der Sicherheitskontrolle durchlaufen hatte, nimmt sie sich Zeit im Bereich des Gates für den Kauf einer Zeitschrift und ein Essen in einem nahegelegenen Restaurant. Die letzte Stunde bis zum Zeitpunkt des Boardings verbringt sie daraufhin mit dem Aufenthalt in einem weiteren Shop sowie im Wartebereich vor dem Gate. Diese Journey ist stellvertretend für viele der beobachteten Journeys.

**Figure 2:**
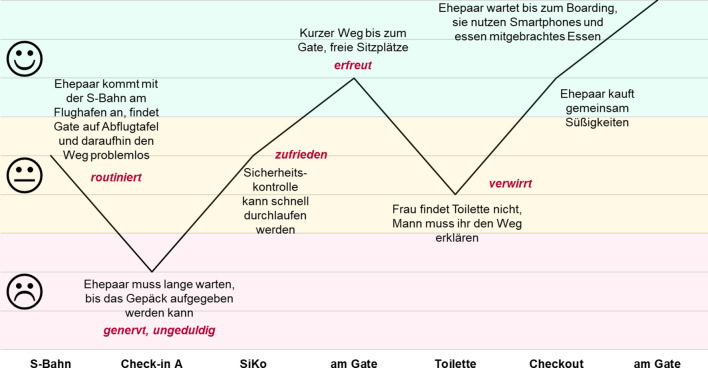

Um die emotionale Entwicklung während der Aufenthalte am Flughafen zu visualisieren, sind sogenannte Emotional-Journey-Diagramme hilfreich. Mit deren Hilfe wird die Stimmung eines Passagiers im Zeitverlauf aufgetragen. Abb. 2 zeigt beispielhaft die Emotional Journey eines Ehepaars, das von München nach Rom flog. Es zeigt sich sehr deutlich, welche Ereignisse am Flughafen typischerweise einen Wechsel der Emotionen auslösen. Während viele Ereignisse routiniert und zufrieden durchlaufen wurden, führten sowohl die lange Wartezeit an der Gepäckabgabe als auch wahrgenommene Schwierigkeiten bei der Suche nach Toiletten zu einem Abschwung der Emotionen. Hingegen stieg die Stimmung, sobald das Gate erreicht werden konnte und sich dort genügend Sitzplätze zur Überbrückung der Wartezeit befanden.

Aus allen Beobachtungen in Kombination mit den geführten Interviews konnten viele übergreifende und auch passagiersegmentspezifische Schlüsse gezogen werden. Dabei konnte einerseits Erwartetes bestätigt, Befürchtungen aber auch entkräftet und Unerwartetes aufgedeckt werden. Viele Beobachtungen bestätigten das sehr hohe Niveau der Kundenzufriedenheit am Flughafen München. Dabei wurden sowohl das moderne Ambiente im Terminal 2, die kurzen Laufwege im Terminal 1 und die Sauberkeit am gesamten Flughafen immer wieder hervorgehoben. Auch Maßnahmen, wie die Beschilderung in arabischer Schrift im Bereich des Terminals 1, in dem besonders viele Reisende aus arabischen Ländern ankommen, wurden als sehr positiv wahrgenommen. Jedoch konnte auch Raum für Verbesserungen identifiziert werden. So zeigten einige Passenger Journeys überraschende Wegverläufe. Beispielsweise ist die herrschende Annahme über Passagiere mit weniger Erfahrung an Flughäfen, dass diese nach Ankunft am Flughafen zuerst möglichst schnell alle notwendigen Stationen wie Gepäckabgabe und Sicherheitskontrolle sowie das Finden des richtigen Gates abarbeiten und sich erst dann entspannen und, sofern genügend Zeit übrig ist, in ein Restaurant oder einen Shop gehen. Dahingegen konnten Passagiere beobachtet werden, die sich ungewöhnlich lange nach der Gepäckabgabe im öffentlichen Bereich aufhielten und sich erst dann auf den Weg zur Sicherheitskontrolle machten, wobei in einigen Fällen die dort dann langen Passagierschlangen das rechtzeitige Erreichen des Gates gefährdeten.

In Bezug auf die Orientierung und Wegefindung, eine der zentralen Fragestellungen im Vorfeld, zeigte sich, dass die Passagiere insgesamt sehr gut mit der Beschilderung zurechtkamen und diese einen wichtigen Beitrag leistet. Jedoch zeigten sich Schwierigkeiten durch teilweise zu klein beschriftete Schilder oder dadurch, dass andere Schilder und Werbebanner das relevante Schild verdeckten. Auch zeigten einige Beispiele die Wichtigkeit des Shadowings. So konnte vermehrt beobachtet werden, dass aufgrund ihrer Größe eigentlich unübersehbare Schilder übersehen wurden, da sie sich an von den Passagieren nicht erwarteten Orten und außerhalb des natürlichen Blickfeldes befanden.

## Digitale Services werden in Flughäfen noch nicht als Standard wahrgenommen

Im Rahmen der Studie wurde auch der vermehrte Einsatz digitaler Services evaluiert. Auch hier zeigten sich in Bezug auf die Wegeführung Schwierigkeiten. Insbesondere Wegangaben auf digitalen Bildschirmen, welche aus Flughafensicht die Vorteile bringen, dynamische und aktuelle Angaben zu zeigen, wurden häufig übersehen, da die Passagiere mit „analogen“ Schildern rechnen, auf Bildschirmen lediglich Werbeanzeigen erwarten und diese somit aus ihrem Blickfeld ausblenden. Auf der anderen Seite kamen digitale Informationsterminals sehr gut an, an denen Passagiere gezielt nach dem gewünschten Ort suchen und sich den individuellen Weg von der aktuellen Position aus anzeigen lassen können.

Auch wurden digitale Services wie Check-in-Automaten oder die elektronische Passkontrolle sehr gut angenommen, denn Passagiere schätzten insbesondere die Zeitersparnis in den Prozessen. Jedoch hatten einige Passagiere Schwierigkeiten bei der Bedienung der neuen Geräte und waren dadurch verunsichert. Viele Passagiere – insbesondere jüngere – wünschen sich dennoch vermehrt digitale Services am Flughafen bzw. einen Ausbau der bestehenden, wie beispielsweise der Flughafen-App, um antizipierte oder sogar Live-Informationen zu Wartezeiten an der Sicherheitskontrolle abrufen zu können.

Darüber hinaus können auf Basis der Shadowing-Ergebnisse die (analogen) Touchpoints identifiziert werden, die besonders wichtig für die Passagiere sind. Darauf aufbauend können Konzepte erarbeitet werden, um diese Touchpoints künftig digital zu erfassen und auszuwerten. So kann beispielsweise über eine automatisierte Videoauswertung die Wartezeit an dem besonders wichtigen Touchpoint der Sicherheitskontrolle ermittelt werden. Bei einer zu hohen Wartezeit können dann wiederum Gegenmaßnahmen (zum Beispiel mehr Mitarbeitende) ergriffen werden.

## Learnings und Best Practices des Shadowings

Die Shadowing-Studie am Flughafen München konnte wichtige Erkenntnisse liefern. Diese Learnings und Best Practices gelten nicht nur für den Luftfahrtbereich, sondern sind auch auf andere Branchen übertragbar.

Da das Shadowing sehr aufwendig ist, muss es sehr gut vorbereitet sein. Auch ist es notwendig, sich auf Kundensegmente oder spezifische Fragestellungen zu fokussieren, um hierzu jeweils genügend Beobachtungen erzielen zu können. Zudem ist es wichtig, dass alle relevanten Fachbereiche und Tochtergesellschaften im Voraus in die Vereinbarung des Fokus’ und der Fragestellungen eingebunden sind, um am Ende des Projekts eine hohe Akzeptanz der Erkenntnisse zu erreichen. So wurden am Flughafen München zu Beginn des Projekts Workshops mit den Fachbereichen und Tochtergesellschaften abgehalten, in welchen das Projekt vorgestellt und die Erwartungen der Fachbereiche eingesammelt wurden. Nur so konnte sichergestellt werden, dass das Projekt in Zusammenarbeit mit allen Fachbereichen realisiert und aus den Erkenntnissen im Anschluss gemeinsame Maßnahmen abgeleitet werden konnten.

## Fazit

Im Rahmen eines Projektes am Flughafen München wurde erstmalig eine Marktforschungsstudie mithilfe der qualitativen Shadowing-Methode durchgeführt. Die Methode stellte sich als sehr geeignet dafür heraus, die tatsächlich zurückgelegten Wege, Emotionen und Bedürfnisse der Passagiere herauszufinden. Auch sind solche qualitativen Daten gut dafür geeignet, vorhandene quantitative Daten anzureichern und darauf aufbauend spezifische Maßnahmen abzuleiten.

Das Projekt hat gezeigt, dass Shadowing geeignet ist, um Kundenbedürfnisse besser zu verstehen – vor allem in Branchen, in denen Kunden üblicherweise nicht während der gesamten Passenger Journey im Kontakt mit Mitarbeitern stehen. Demnach ist die Methode auch für andere Transportbereiche, wie beispielsweise Bahnhöfe interessant, aber auch große Räumlichkeiten wie in einem Supermarkt könnten einen weiteren Anwendungsbereich für Shadowing darstellen.
